# Eph receptors: the bridge linking host and virus

**DOI:** 10.1007/s00018-019-03409-6

**Published:** 2019-12-31

**Authors:** Jia Wang, Xiang Zheng, Qiu Peng, Xuemei Zhang, Zailong Qin

**Affiliations:** 1 Department of Immunology, Changzhi Medical College, Changzhi, 046000 Shanxi China; 2 Department of Pathology, Affiliated Hospital of Guilin Medical University, Guilin, 541001 Guangxi China; 3 School of Basic Medical Science, Cancer Research Institute, Central South University, Changsha, 410008 Hunan China; 4 Laboratory of Genetics and Metabolism, Maternal and Child Health Hospital of Guangxi Zhuang Autonomous Region, Guangxi Birth Defects Research And Prevention Institute, Nanning, 530003 Guangxi China

**Keywords:** Eph receptor, Virus infection, Virus-associated cancer, EBV, KSHV

## Abstract

Eph (erythropoietin-producing hepatoma) receptors and Ephrin ligands constitute the largest subfamily of receptor tyrosine kinase (RTK), which were first discovered in tumors. Heretofore, Eph protein has been shown to be involved in various tumor biological behaviors including proliferation and progression. The occurrence of specific types of tumor is closely related to the virus infection. Virus entry is a complex process characterized by a series of events. The entry into target cells is an essential step for virus to cause diseases, which requires the fusion of the viral envelope and host cellular membrane mediated by viral glycoproteins and cellular receptors. Integrin molecules are well known as entry receptors for most herpes viruses. However, in recent years, Eph receptors and their Ephrin ligands have been reported to be involved in virus infections. The main mechanism may be the interaction between Eph receptors and conserved viral surface glycoprotein, such as the gH/gL or gB protein of the herpesviridae. This review focuses on the relationship between Eph receptor family and virus infection that summarize the processes of viruses such as EBV, KSHV, HCV, RRV, etc., infecting target cells through Eph receptors and activating its downstream signaling pathways resulting in malignancies. Finally, we discussed the perspectives to block virus infection, prevention, and treatment of viral-related tumors via Eph receptor family.

## Introduction

**Fig. 1:**
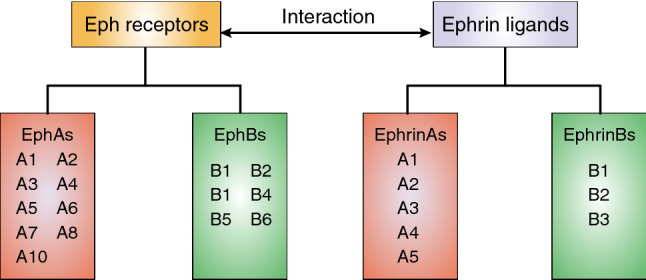
Members of Eph family. In the human genome, there are totally nine EphA and five EphB receptors. The EphA receptors promiscuously bind five glycosylphosphatidylinositol (GPI) linked Ephrin-A ligands, and the EphB receptors promiscuously bind three transmembrane Ephrin-B ligands

Eph (erythropoietin-producing hepatoma) is a big family of receptor tyrosine kinases and plays key roles in physiological and pathological processes in development and disease [1–3]. A total of 14 Eph receptors have been found in humans, which can be subdivided into two subfamilies including EphA and EphB (Fig. 1) based on amino acid sequence homology and relative binding affinities to glycosylphosphatidylinositol (GPI) linked Ephrin-A or transmembrane Ephrin-B ligands [4, 5]. There are nine EphA receptors, which promiscuously bind five Ephrin-A ligands, and five EphB receptors, which promiscuously bind three Ephrin-B ligands [6]. Given Eph receptors and their ligands are often overexpressed in human malignancies and associated with poor prognosis, Eph receptors and Ephrins are considered as very promising drug targets [7, 8].

Virus infection is closely related to the occurrence and development of many diseases. In recent years, many studies have identified the relationship between virus infection and tumors. Well-known virus-related tumors include: (1) EBV-positive lymphoma, nasopharyngeal carcinoma, and gastric cancer [9–11], (2) Kaposi’s sarcoma-associated herpesvirus (KSHV) in Kaposi’s sarcoma (KS) [12], primary effusion lymphoma (PEL) [13], and multicentric castleman’s disease (MCD) [14], (3) HBV and HCV in liver cancer, etc. [15].

Virus infection of the host involves a complex multi-step process. The first step is viral attachment and entry through interaction between viral glycoprotein and receptors on the surface of the host. For example, EBV-infecting epithelial cells mainly rely on the interaction of gH/gL glycoproteins with host surface integrin receptors (αvβ5, αvβ6, αvβ8) [16–18]. KSHV interacts with integrin receptors (α3β1, αvβ3, α5β5, α9β1) on the surface of epithelial cells and fibroblasts through the encoded gB glycoprotein to facilitate its entry [19]. In addition, HCV entry into the target cells is mediated through binding of HCV envelope glycoproteins to glycosaminoglycans involving viral envelope glycoproteins as well as several cellular attachments and entry factors [20, 21] including CD81 [22], scavenger receptor class B type I (SR­BI) [23], claudin­1(CLDN1) [24], and occludin (OCLN) [25].

Integrin family is well known as an entry receptor for most herpes viruses. However, in recent years, some studies have reported that the Eph receptors family can also act as an entry receptor-mediating infection of pathogenic microorganisms. Given the tyrosinase properties of Eph receptors, there have been a large number of small-molecule inhibitors targeting Eph receptors, which provide a valuable opportunity for the treatment and prevention of Eph receptor-associated virus infection. In this review, we focus on the relationship between Eph receptor and virus infection and discuss the possibility of targeting Eph receptor signaling pathways as alternative antivirus therapeutic strategies.

## Structure and function of Eph family

### Structure of Eph and Ephrin families

**Fig. 2:**
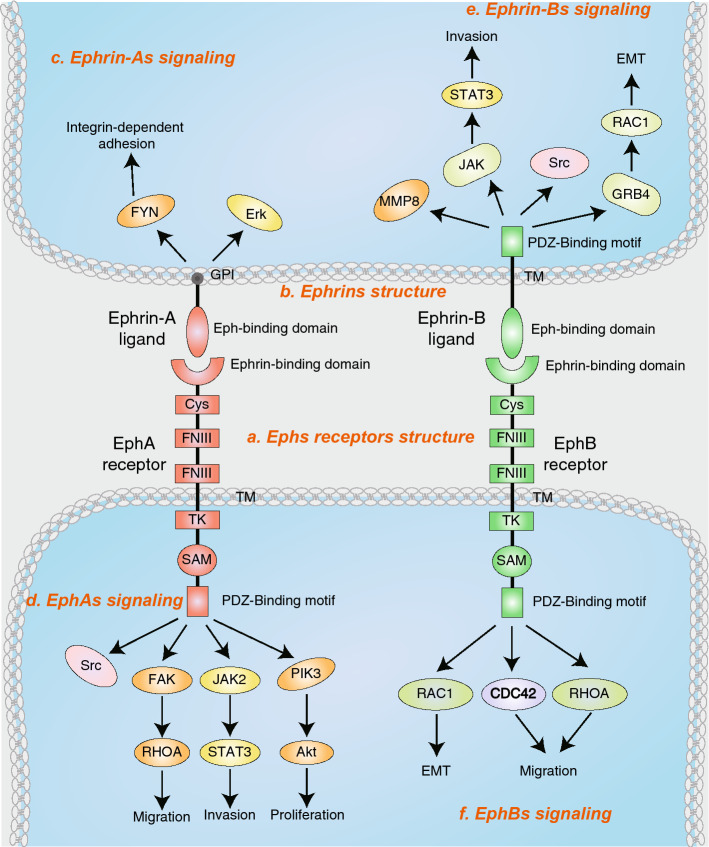
Domain structure and signaling concepts of Ephs and Ephrins. **a** Eph receptors (Ephs) consist of a ligand-binding domain (LBD), cysteine-rich region (Cys), two fibronectin III repeat (FNIII), a transmembrane region (TM), a juxtamembrane region (JM), a tyrosine kinase domain (TK), a sterile alpha motif (SAM) a PSD-95/Dlg/ZO-1. GPI and glycosylphosphatidylinositol binding motif (PDZ). **b** Ephrin-As are linked to the membrane via a glycosylphosphatidylinositol (GPI) moiety, Ephrin-Bs are anchored by a transmembrane domain and contain a cytoplasmic tail. **c** Ephrin-A signaling promotes activation of FYN, and Erk. **d** EphA receptors directly activate Src and RHOA through focal adhesion kinase (FAK). EphA receptors activate JAK2 by STAT3 (signal transducer and activator of transcription 3). EphA2 activates Akt in pancreatic cancer cells. **e** Ephrin-Bs promote EMT and invasion by activating Src, STAT3, MMP8 (matrix metalloproteinase 8), and RAC1. **f** EphBs activate RHOA, RAC1, and CDC42 which promote cancer cell migration and invasion

The Eph receptor consists of three parts [6]: (1) extracellular domain, including a ligand-binding domain, a cysteine-rich domain, and two fibronectin type III repeats, (2) transmembrane domain, (3) intracellular domain, consisting of a juxtamembrane region, a tyrosine kinase domain, a sterile alpha motif (SAM), and a C-terminal PSD95/discs large/zona occludens 1 protein (PDZ)-binding motif. The Ephrin-A ligands, unlike the Eph receptor, have no intracellular domain and are anchored on the membrane by the glycosyl lipoinositol (GPI) group. Ephrin-B ligands have a hydrophobic transmembrane region and a short intracellular region (Fig. 2).

### Signaling modes of Ephs and Ephrins

Binding of Eph receptor to Ephrin proteins in adjacent cells produces cell-dependent bi-directional signaling that regulates cell shape, movement, survival, and proliferation [3, 7, 8]. The Eph forward signaling is dependent on binding to the ephrin proteins, which can undergo clustering, auto-phosphorylation, and activation of kinase activity. Many studies have reported that Eph receptor’s forward signaling could activate Src, RHOA, RAC1, CDC42, STAT3 (signal transducer and activator of transcription 3), and PIK3/Akt in a variety of tumors which promote cancer-cell migration and invasion [26–31]. In addition, binding of Eph receptors to Ephrin proteins can also lead to endocytosis and proteolysis [8, 32–35]. The Ephrin reverse signaling is activated by interacting with the Eph receptor. Ephrin-As transduct signaling via glycosylphosphatidylinositol groups interacting with transmembrane partners. However, signaling transduction of Ephrin-Bs is involved in tyrosine and serine phosphorylation by associating transmembrane structure of Ephrin-Bs with various effector proteins [8, 36]. Some studies have reported that Ephrin reverse signaling could promote EMT and invasion in a variety of tumor cells by activating Src, STAT3, MMP8 (matrix metalloproteinase 8), and RAC1 [30]. Given the involvement of Eph in multiple life processes and their roles in cancer progression, researchers have conducted intensive research on the function of the Eph family over the past few decades. Moreover, several studies have shown that Eph family is closely related to virus infection in recent years (Table 1).

**Table 1:** Ephs involved in virus entry

Virus	Entry receptors	Binding virus glycoprotein	Target cells	Refs.
EBV	EphA2	gH/gL and gB	Human gastric adenocarcinoma cell (AGS), human embryonic kidney epithelial cells (HEK293)	[66, 67]
KSHV	EphA2	gH/gL	Human embryonic kidney epithelial cells (HEK293T)	[81, 85]
HCV	EphA2	E2 (HCV envelope glycoprotein)^a^	Human hepatocarcinoma cell (Huh7.5.1)	[93]
RRV	EphA4, EphA5, EphA7, EphB2, EphB3	gH/gL	B cells and endothelial cells	[102, 104]

^a^Not confirmed

### Function of Ephs and Ephrins in malignancies

Many studies have verified Ephs and Ephrins, aberrantly expressed in tumors which can drastically affect malignancy including progression, metastatic spread, and patient survival [30, 37]. Ephs and Ephrins expression can increase or decrease during cancer progression caused due to transcriptional regulation by oncogenic signaling pathways, promoter methylation, and microRNAs [38, 39]. EphA2, EphB2, and EphB4 are the Eph receptors that most widely deregulated expression in tumors [40–42]. EphA2 is frequently overexpressed in melanoma, glioma, breast cancer, prostate cancer, lung cancer, cervical cancer, colon cancer, esophageal cancer, gastric cancer, ovarian cancer, bladder cancer, and renal-cell carcinomas [42, 43]. Many studies have shown that overexpression of EphA2 is closely related to the activation of some tumor-associated signaling pathways, such as Wnt/β-catenin pathway, Ras/MAPK pathway, and Akt/mTOR pathway [44–46]. Some mutations of Eph receptors are predicted to play a vital role in cancer progression. For example, EphB2 mutations have been identified in human gastric, colorectal, and prostate tumors, some of which can impair kinase function [47–50]. Furthermore, EphB2 has been shown to be upregulated in glioblastoma as a consequence of decreased miR-204, which led promote invasiveness [51, 52]. Another Eph receptor, EphB4, the interaction partners of Ephrin-B2, is a prominent marker of normal and tumor vasculature [53]. In addition, EphB4 can trigger RAS–MAPK-dependent proliferation of MCF7 cancer cells which are considered to be prognostic for poor survival of patients with breast cancer [54, 55].

## Role of Eph family in virus infection

### EphA2 is an EBV entry receptor

EBV is a ubiquitous human gamma-herpes virus that is present in over 90% of adults worldwide. EBV is closely related to nasopharyngeal carcinoma, 10% of gastric cancer, and various B-cell lymphomas. EBV is associated with B cells and epithelial malignancies, indicating that its main tropism is the infection of B cells and epithelial cells [56]. Human B cells are the primary target of EBV infection. The mechanisms of EBV entering B cells have been well documented. EBV infects B cells requiring interaction between its glycoproteins gp350 or splicing mutant gp220 and the cell surface receptor CD21, and interaction of viral glycoproteins gp42 with MHC II [57, 58]. It is generally believed that activated-gp42 is able to transduce signals to gH/gL and gB to prevent it from interacting with other cellular receptors, including integrin receptors. Therefore, gp42 is considered to be indispensable for EBV infecting B cells specifically [59–61].

Unlike B cells, EBV entry into epithelial cells is independent of gp350/gp220 and gp42, while glycoproteins gH/gL and gB are required for EBV infection of epithelial cells [62–65]. Studies from Chesnokova et al. have shown that the interaction of glycoprotein gH/gL with three epithelial integrin receptors (αvβ5, αvβ6, αvβ8) is associated with EBV entry into epithelial cells [16, 18]. Recently, Zhang et al. found that Ephrin receptor A2 (EphA2) was associated with EBV entry into epithelial cells through microarray and RNAi library screening. Knockdown of EphA2 by siRNA or CRISPR-Cas9 can significantly reduce EBV infection of epithelial cells, while overexpression of EphA2 can restore the EBV infection of epithelial cells [66]. Further studies found that the interaction of EphA2 and EBV-encoded proteins gH/gL and gB can promote the fusion and internalization of EBV, and the Ephrin ligand binding domain and fibronectin domain of EphA2 arerequired for EphA2-mediated EBV infection. These results indicate that EphA2 is critical for EBV entry into epithelial cells [66]. Chen et al. also found that EphA2, but not the extracellular region of EphA4, interacts with EBV-encoded gH/gL to promote EBV fusion and endocytosis [67]. Moreover, they show that the integrin receptors αvβ5, αvβ6, and αvβ8 have no effect on the entry of EBV into epithelial cells, which is distinct from the findings of Chesnokova et al. [67]. The discovery of EphA2 as a novel EBV-infected epithelial cell receptor is of great significance and may uncover new attractive targets, which could be used to develop new intervention strategies for blocking EBV infection.

Although EphA2 is required for EBV infection of epithelial cells, EBV-encoded proteins also regulate the expression of Eph family. Huang et al. found that EphA4 expression was down-regulated in EBV-positive diffuse large B-cell lymphoma and correlated with patient prognosis. Mechanism studies found that down-regulation of EphA4 expression is mainly caused by the regulation of ERK-SP1 signaling pathway by EBV-encoded LMP1 protein. These findings suggest that EphA4 may be a potential therapeutic target for diffuse large B-cell lymphoma [68]. In addition, Zhao et al. reported that there were six hypermethylated genes in EBV-positive gastric cancer cells (AGS-EBV), including EphB6 [69]. In contrast, Xiang et al. found that the expression level of EphA2 was significantly higher in EBV-positive nasopharyngeal carcinoma cells than in EBV-negative nasopharyngeal carcinoma cells (CNE2-EBV vs. CNE2) using RNA-seq analysis. In NPC samples, the upregulation of EphA2 expression in EBV-positive NPC samples was further confirmed. Mechanistically, PI3K/Akt signaling pathway is significantly activated in both xenografts and clinical samples of NPC and EBVaGC [70]. Miao et al. reported that a reciprocal regulatory loop between EphA2 and Akt, which was characterized by unligated EphA2 was a substrate for Akt and negatively regulated by the ligand-activated EphA2 in turn [71]. These findings suggest that EBV may regulate the expression of EphA2 through the AKT signaling pathway. Furthermore, Kim et al. also reported EphA2 and EBV-associated gastric tumor cells were involved in the formation of vasculogenic mimicry (VM) channels [72]. Interestingly, EphA2 expression was not detectable in B-cell lymphoma cell lines (Akata, Akata-EBV and Raji) susceptible to EBV infection. These findings suggest that EphA2 is essential for EBV infection of epithelial cells, but not required for B cell infection [66].

### EphA2 is a KSHV entry receptor

Kaposi’s sarcoma-associated herpesvirus (KSHV) or human herpesvirus-8 (HHV-8) was first isolated from patients with AIDS-related Kaposi’s sarcoma (AIDS-KS) in 1994 by Chang et al. [73]. KSHV is a tumor-associated virus that is the causative agent of Kaposi’s sarcoma (KS), primary effusion lymphoma (PEL), and multicentric castleman’s disease (MCD) [73–75]. The KSHV genome is highly consistent with γ-1 Epstein-Barr virus (EBV), γ-2 herpesvirus saimiri (HVS), and rhesus r virus (RRV). Similar to all members of the herpesvirus family, KSHV has a double-stranded DNA genome (~ 160 kb) packaged in the capsid, and the capsid is surrounded by a lipid envelope containing five conserved glycoproteins [76, 77]. The KSHV genome encodes more than 100 open reading frames (ORFs), of which 4–75 are classified according to their homology to the HVS ORF [78]. KSHV has a wide range of cellular tropism, and it can infect various target cells in vitro and in vivo. KSHV entry and signal transduction are complex events that vary greatly depending on the host cell [79]. Moreover, KSHV can form a variety of different internalization pathways into the host through different combinations of host cell surface receptors [79]. Studies have shown that multiple KSHV glycoproteins are involved in binding to host cell membranes and are involved in interacting with surface receptor of host cells, thereby inducing a cascade of signaling pathways to promote endocytosis. Subsequent steps include fusion of the viral envelope with the endosomal membrane, release of the viral capsid in the cytoplasm, and transport of KSHV DNA into the nucleus. These processes are essential for the virus infection, which relies on intricate intermolecular interactions.

Ephrins have been reported to regulate macropinocytosis and clathrin-dependent endocytosis in a variety of cells [3, 80]. A recent study reported that the interaction of EphA2 and KSHV glycoprotein gH/gL could promote virus entry [81]. Pretreatment of target cells with soluble EphA2 ligand or incubation of KSHV virions with soluble EphA2 protein inhibited KSHV infection [81]. Knockdown of EphA2 significantly reduced the entry of KSHV, while overexpression of EphA2 increased the entry of KSHV [81]. Significantly, examined EphA2 expression in tissue sections from individuals with Kaposi’s sarcoma using quantitative RT-PCR and in situ histochemistry showed a strong correlation between EphA2 expression and KSHV infection both in cultured Kaposi’s sarcoma-derived cells and in Kaposi’s sarcoma tissues [47]. Previous studies have shown that KSHV-induced ERK and NF-κB signaling pathways are essential for the initiation of viral or host gene expression [82–84]. Interestingly, the MAPK pathway can control the expression of the EphA2 receptor [45]. These results explain, at least in part, the reason why the expression of EphA2 is strongly correlated with KSHV infection. Moreover, binding of gH/gL to EphA2 would induce phosphorylation of EphA2 and promote the internalization of KSHV. These findings indicate that EphA2 is a specific entry receptor for KSHV infection [81].

Chakraborty et al. also reported that EphA2 bind to FAK, Src, and other signaling molecules in a lipid-raft to form a signal complex, which could promote KSHV entry into macropinosomes [85]. Another group reported that EphA2 played a crucial role in the coordination and amplification of KSHV-induced signaling in fibroblasts and the virus is endocytosed by the clathrin-mediated endocytic pathway [86]. Interestingly, a recent study found that androgen receptors could also promote KSHV-infected host cells by interacting with EphA2, which revealed why KSHV had a higher infection ratio in male than female [87].

### EphA2 is a co-factor for hepatitis C virus entry

HCV was originally isolated from serum in 1989, and its genome is a single-stranded RNA of approximately 9 kb [88]. HCV is the leading cause of cirrhosis and hepatocellular carcinoma. Although newly developed antiviral drugs targeting HCV proteins have been shown to increase virological response, there are still some toxicity and resistance [89]. HCV entry is a multi-step process, which is mainly mediated by viral envelope glycoproteins, adhesion proteins, and entry factors of cell surface [90]. The attachment of the virus to the target cells is mediated by the binding of the HCV envelope glycoprotein to the glycosaminoglycan [91]. The involvement of CD81 is essential for HCV in the process of clathrin-dependent endocytosis [92]. Joachim Lupberger et al. screened EphA2 and epidermal growth factor receptor (EGFR) as co-receptor factors to facilitate HCV entry using RNAi kinase libraries [93]. Small molecule inhibitors targeting tyrosine kinase can significantly attenuate HCV infection (55). Mechanistic studies indicate that EphA2 and EGFR mediate HCV entry by modulating the interaction of the CD81-claudin-1 (CLDN1) co-receptor with viral glycoproteins [93]. EphA2 has been reported highly expressed in human liver. Cui et al. found that EphA2 expression was prominent in highly invasive hepatoma cells, and its overexpression was significantly correlated with decreased differentiation and poor survival for HCC patients [94]. In addition, Lee et al. identified that high expression of EphA2 was related to lymph node metastasis in 32 human hepatocellular carcinoma patients, all of these patients were infected with HCV or HBV [95]. However, direct evidence of whether the malignant phenotype of liver cancer through control the expression of EphA2 caused by HCV was not reported. These results demonstrate that EphA2 can act as new co-receptors for HCV entry and indicate that tyrosine kinase inhibitors have significant antiviral effects. Therefore, inhibition of EphA2 may be a new strategy for preventing and counteracting HCV infection.

### EphAs and EphBs are rhesus monkey rhadinovirus (RRV) entry receptors

Rhesus monkey is a primate species that is genetically and physiologically similar to humans. Scientists have decoded the genome of the rhesus monkey and compared it with humans. Nucleotide sequences that aligned between the humans and rhesus monkey averaged at 93.54% identity [96]. The γ-2 herpesvirus is a unique subfamily of the lymphotropic herpesviruses. Rhesus monkey rhadinovirus (RRV) is a natural infectious agent with a high frequency of infection in both feeding and wild rhesus monkey populations [97]. RRV is a rhesus monkey ortholog of the human Kaposi’s sarcoma-associated herpesvirus (KSHV, human herpesvirus-8, HHV-8) [98, 99]. RRV has been reported to be associated with B-cell malignancies and is similar to B-cell malignancies caused by KSHV [100–103]. Hahn et al. reported the gH/gL glycoprotein complex of rhesus monkey rhadinovirus could bind to the virus and mediate its entry into target cells via cellular Ephrin receptor tyrosine kinase proteins [104]. Unlike KSHV, which enters into host cells through EphA2 receptor mainly, RRV can utilize more type A and B Eph receptors as entry receptors (10 of 14 Eph receptors can interact with gH/gL glycoprotein complexes) [104]. Furthermore, RRV entry into B cells and endothelial cells is almost entirely dependent on the Eph receptor pathway, whereas RRV entry into fibroblasts and epithelial cells is via the Eph receptor-independent pathway [100]. Therefore, it suggests that KSHV may also infect host cells through Eph receptor-independent pathways in some cases.

### Eph family as an entry receptor for other viruses

Although the Eph family has been widely involved in virus infections such as EBV, KSHV, and HCV, the role of Eph receptors and Ephrins in other virus infections remain to be discovered. For example, Karlas et al. found that EphB6 played an important role in H1N1 influenza virus entry and replication using siRNA library screening in A549 lung cancer cell [105]. Xu et al. reported that the expression patterns of Ephrin-B2 and Ephrin-B3 might be the reason of acute lymphatic necrosis caused by henipavirus infection [106]. Moreover, Ephrin-B2 and Ephrin-B3 promote the entry mechanism of henipavirus mainly by interacting with the viral G protein to activate the viral F protein, thereby inducing fusion of the viral capsid and the host cell membrane [107]. In the study by Dewannieux et al. the mouse IAPE (Intracisternal A-type Particles elements with an Envelope) family was able to utilize five Ephrin-A family members, such as Ephrin-A4 as entry receptor to infect host cell. Interestingly, Ephrin-B family with higher homology to Ephrin-A family do not mediate IAPE entry [108]. In recent years, Eph family has also been reported as entry receptor for other pathogenic microorganisms. Swidergall et al. found that EphA2 could act as a pattern recognition receptor to promote fungal infection of host cells by interacting with fungal surface glycoproteins [109].

## Role of Eph family in antiviral therapies

The Eph family has been increasingly recognized as an attractive therapeutic target for many diseases, ranging from anticancer therapeutics to modulators of synaptic plasticity, bone homeostasis, and remodeling and stem cell biology [110–112]. Some therapeutic approaches have been developed to modulate Eph–Ephrin function, including small-molecule kinase inhibitors, Ephrin-mimetic peptides, short hairpin RNAs, and monoclonal antibodies [113–116]. The involvement of Eph family in virus infection provides new strategies for antiviral therapies. Zhang et al. reported that soluble EphA2 protein, antibodies against EphA2, soluble EphA2 ligand Ephrin-A1, or the EphA2 inhibitor 2,5-dimethylpyrrolyl benzoic acid could efficiently block EBV epithelial cell infection [66]. Xu et al. found that Ephrin-B2’s extracellular domain (ECD) and an antibody to the henipavirus, glycoprotein that bind Ephrin-Bs had antiviral activity [106]. In addition, EphA2/Ephrin-A ECDs and EphA2-targeting antibodies have also been successfully used to inhibit KSHV and HCV of cultured cells [81, 85, 93]. These studies demonstrated that suitable targeting of Eph–Ephrin function holds considerable promise for antiviral therapies.

## Conclusions

**Fig. 3:**
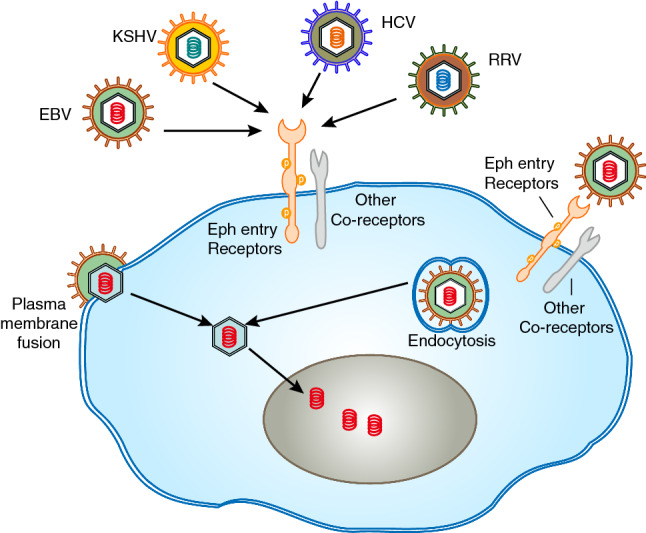
Interaction of virus and Ephs is crucial for viral entry. A generic virus is shown binding to Ephs entry receptors and co-receptors (integrins). The virion interaction with entry receptors triggers specific entry pathways, two examples are shown. Fusion at the plasma membrane mediated by entry receptors (e.g., Epstein–Barr virus infection of epithelial cells). Endocytosis mediated by entry receptors (e.g., Kaposi’s sarcoma-associated herpesvirus infection of multiple cell types)

Virus infection is closely related to the occurrence and development of many diseases, posing a great threat to human life and health. Antiviral therapies are currently limited by drug resistance, toxicity, and high cost. Therefore, new antiviral prevention and treatment strategies are imminent. The mechanism by which viral entry involves a range of events, including interactions with target cell surface receptors, endocytosis, and nuclear release (Fig. 3). Host cell surface receptors are key molecules for viral recognition and are involved in linking viruses to host cells. Therefore, there is a significant potential for the preparation of antiviral drugs for specific target cell receptors.

Current research has found that Eph family is involved in the entry of multiple viruses, and because of the involvement of Eph–Ephrin in many biological processes, there are already a large number of approved targeted inhibitors against the Eph family. These provide a rich opportunity for intervention and treatment for virus infection.
